# Red Orange and Lemon Extract Ameliorates the Renal Oxidative Stress and Inflammation Induced by Ochratoxin A through the Modulation of Nrf2

**DOI:** 10.3390/toxins16030151

**Published:** 2024-03-14

**Authors:** Consiglia Longobardi, Sara Damiano, Simona Fabroni, Serena Montagnaro, Valeria Russo, Emanuela Vaccaro, Antonio Giordano, Salvatore Florio, Roberto Ciarcia

**Affiliations:** 1 Department of Veterinary Medicine and Animal Productions, University of Naples Federico II, Via F. Delpino n.1, 80137 Naples, Italy; consiglia.longobardi@unina.it (C.L.); serena.montagnaro@unina.it (S.M.); valeria.russo@unina.it (V.R.); emanuela.vaccaro@unina.it (E.V.); salvatore.florio@unina.it (S.F.); 2 Council for Agricultural Research and Economics (CREA), Research Centre for Olive, Fruit and Citrus Crops, C.so Savoia n.190, 95024 Acireale, Italy; simona.fabroni@crea.gov.it; 3 Sbarro Institute for Cancer Research and Molecular Medicine, Center of Biotechnology, College of Science and Technology, Temple University, Philadelphia, PA 19122, USA; antonio.giordano@unisi.it; 4 Department of Medical Biotechnologies, University of Siena, 53100 Siena, Italy

**Keywords:** RLE, kidney, OTA toxicity, Nrf2, ROS

## Abstract

Background: The presence of ochratoxin A (OTA) in food and feed is a public health concern. OTA intoxication is caused by several mechanisms, one of which consists of the alteration of the antioxidant activity of the cell due to the oxidative stress (OS). In this context, the use of natural antioxidant substances could be a potential biological decontamination method of mitigating the negative outcomes induced by OTA. Methods: we aimed to investigate how a red orange and lemon extract (RLE), rich in anthocyanins, would affect OTA-treated rats. The current work sought to clarify the renal protective efficacy of RLE in an OTA-treated rat model (RLE (90 mg/kg b.w.); OTA (0.5 mg/kg b.w.)) by investigating, thorough Western blot analysis, the involvement of the Nuclear factor erythroid 2-related factor 2 (Nrf2) pathway. The OS parameters and inflammatory status were evaluated by spectrophotometry. The inflammatory infiltrates in the kidney were evaluated by immunohistochemical assays. Results and Conclusion: Our findings showed a significant increase in oxidative and inflammatory parameters after OTA exposure, while the OTA + RLE co-treatment counteracted both the inflammatory and OS damage through the modulation of the Nrf2 pathway.

## 1. Introduction

Ochratoxin A (OTA), a mycotoxin produced by several fungi such as *Aspergillus* and *Penicillium*, is known to cause organ damage in several animal species [1,2,3]. Evidence in the literature suggests that it also causes hepatotoxicity, neurotoxicity, genotoxicity, and carcinogenicity in both animals and humans [4,5]. OTA and its metabolites reduce the glomerular filtration rate, impair cellular immunity and antioxidant status, and enhance reactive oxygen species (ROS) generation in kidney [4,6]. OTA induces toxicity in several organs in rodents and several animal species [7,8], but the kidney remains the primary target organ [8].

The harmful effects of OTA involve a variety of mechanisms, one of which is the alteration of the antioxidant activity in cells [9] with the induction of oxidative stress (OS) [10], a pathogenic state caused by the imbalance between antioxidants and oxidant molecules that can lead to cell organelle damage by inducing glutathione (GSH) depletion, apoptosis, and ROS production [10]. Moreover, ROS activate Nuclear factor type kB (NF-κB), activator protein-1 (AP-1), and Transforming Growth Factor-β (TGF-β) [11,12]. Various studies have identified OTA as an inhibitor of Nuclear factor erythroid 2-related factor 2 (Nrf2), a transcription factor responsible for the regulation of cell protection and antioxidant activation [13]. Nrf2 is physiologically involved in many cellular events, such as inflammation, apoptosis, cell survival, angiogenesis, and innate and acquired immunity. It has been well demonstrated that when OTA affects the antioxidant defense system, modulation in Nrf2 expression occurs. Therefore, the activation of the Nrf2 during OS disease regulates the expression of genes involved in the inflammatory and OS processes [13,14], influencing the expression of related genes [15].

Most ingredients used in animal feed formulation come from grains and plants, which are easily contaminated by mycotoxins like OTA [16]. As a result, OTA transmission and build up in the food chain might result in the accumulation of biotoxicity in both humans and animals [9]. OTA exhibits a possible human toxicological risk. In fact, it is suspected to be a cause of Balkan endemic nephropathy (BEN) [17] and chronic interstitial nephropathy (CIN) [18]. The International Agency for Research on Cancer (IARC) classified OTA as a Group 2B human carcinogen [8], although its carcinogenicity in humans has not been fully correlated. As a result, worldwide restrictions have been established to minimize the introduction of contaminated spices into human and animal feed and food, and the European Commission (EC) has established an OTA maximum tolerable limit (MTL) [19,20].

Based on this evidence, the problem of food and feed contamination has raised to the level of a public health issue that must be managed and monitored over time. Given the difficulties of preventing OTA contamination, the scientific community plays an important role in developing innovative food decontamination procedures. Many strategies of OTA decontamination are used, including physical, chemical, and biological treatments, which come with their own disadvantages and advantages [21,22,23]. Among the latter, biological decontamination has been widely used for the removal of mycotoxins due to its potential to eliminate their cancerogenic and toxic profiles.

Several studies have been conducted using edible plant extracts. Among the most important bioactive molecules identified in the polyphenol family, anthocyanins are certainly the compounds with the most marked beneficial properties, such as antioxidant, anti-inflammatory, and antiviral activity [24,25,26]. Therefore, this study aimed to investigate the antioxidant and anti-inflammatory effects of a red orange and lemon extract (RLE) containing anthocyanins in an OTA-treated animal model. In particular, we sought to clarify the renal protective effect of RLE in OTA-treated rats by investigating the involvement of Nrf2.

## 2. Results

### 2.1. RLE Suppressed OTA–Kidney Oxidative Stress

**Figure 1:**
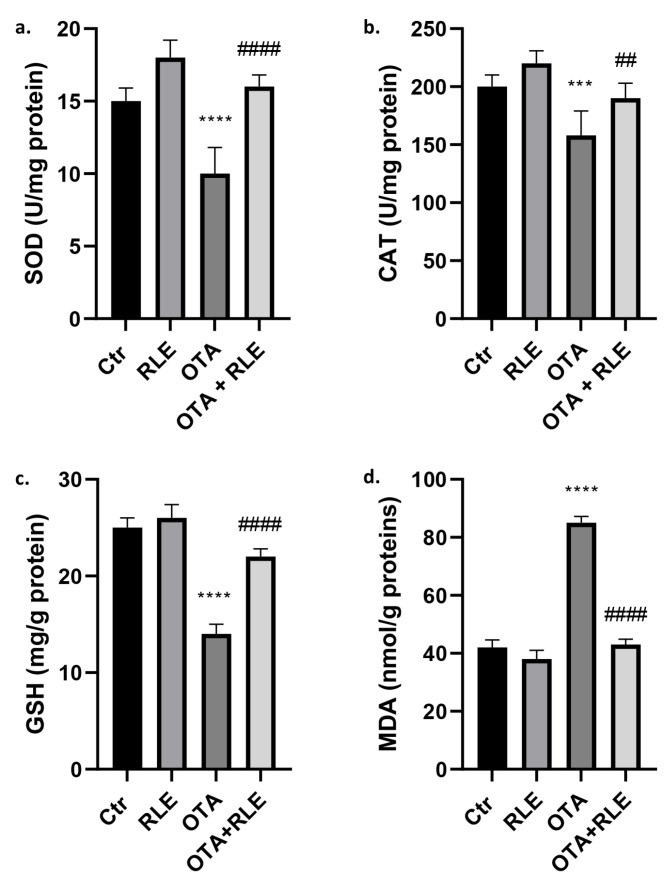
Effect of red orange and lemon extract (RLE) on kidney of ochratoxin A (OTA)-intoxicated rats. (**a**) Superoxide dismutase (SOD), (**b**) catalase (CAT), (**c**) glutathione (GSH), and (**d**) malondialdehyde (MDA) levels. Control (Ctr) group; RLE group (90 mg/kg b.w.); OTA group (0.5 mg/kg b.w.); OTA plus RLE group. Data are reported as mean ± SD (*n* = 6 rats/group) (*** *p* < 0.001 and **** *p* < 0.0001 vs. Ctr; ## *p* < 0.01 and #### *p* < 0.0001 vs. OTA).

Figure 1a–c show the levels of superoxide dismutase (SOD), catalase (CAT), and GSH in the kidney of OTA-treated animals. The results showed that the levels of SOD, CAT, and GSH in the kidneys of the OTA group were significantly reduced compared to the Ctr group (*p* < 0.001 and *p* < 0.0001). In the rat treated with RLE plus OTA (OTA + RLE), we observed a restoration in antioxidant parameters (*p* < 0.01 and *p* < 0.0001), with values similar to the Ctr group.

Furthermore, a significant increase in malondialdehyde (MDA) was observed (*p* < 0.0001) in the OTA group compared to the Ctr group. RLE co-treatment (OTA + RLE) restored this value (*p* < 0.0001) (Figure 1d).

### 2.2. RLE Prevented Kidney Inflammatory Alteration

**Figure 2:**
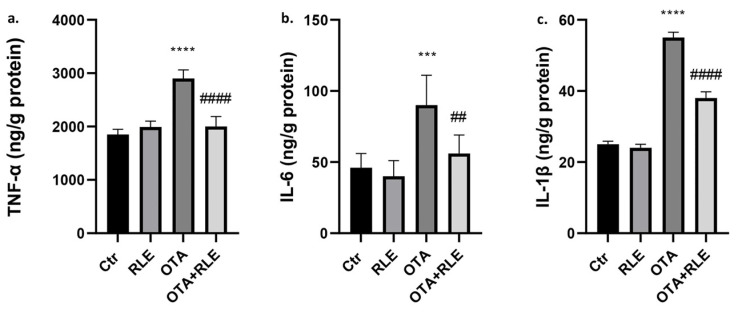
Effect of red orange and lemon extract (RLE) on the kidney of ochratoxin A (OTA)-intoxicated rats. (**a**) Tumor necrosis factor-alpha (TNF-α), (**b**) interleukin-6 (IL-6), and (**c**) interleukin 1β (IL-1β) level. Control (Ctr) group; RLE group (90 mg/kg b.w.); OTA group (0.5 mg/kg b.w.); OTA plus RLE (OTA + RLE) group. Data are reported as mean ± SD (*n* = 6 rats/group) (*** *p* < 0.001 and **** *p* < 0.0001 vs. Ctr; ## *p* < 0.01 and #### *p* < 0.0001 vs. OTA).

As shown in Figure 2, tumor necrosis factor alpha (TNF-α), interleukin (IL)-1β, and IL-6 were increased in the OTA-treated group compared to untreated animals (*p* < 0.001 and *p* < 0.0001). However, co-administration with RLE modulated all these values compared to the OTA-treated group (*p* < 0.01 and *p* < 0.0001). No statistical differences in TNF-α, IL-1β, and IL-6 levels were found between Ctr and RLE groups.

### 2.3. Kidney Tissue Levels of NO and iNOS

**Figure 3:**
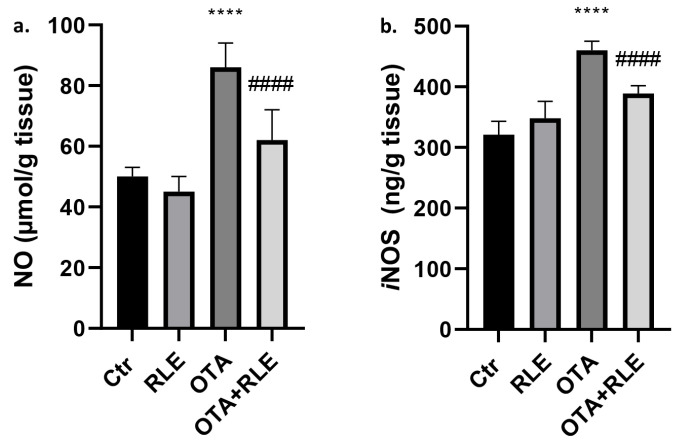
Effect of red orange and lemon extract (RLE) on kidney tissue of ochratoxin A (OTA)-intoxicated rats. (**a**) Nitric oxide (NO) and (**b**) inducible nitric oxide synthase (*i*NOS) levels. Control (Ctr) group; RLE group (90 mg/kg b.w.); OTA group (0.5 mg/kg), and OTA plus RLE (OTA + RLE). Data are reported as mean ± SD (*n* = 6 rats/group) (**** *p* < 0.0001 vs. Ctr; #### *p* < 0.0001 vs. OTA).

Nitric oxide (NO) parameters in the kidney are shown in Figure 3a. In the OTA-treated group, the NO levels were higher compared to the Ctr group (*p* < 0.0001). Moreover, RLE administration significantly restored the production of NO in OTA-treated rats (*p* < 0.0001). No significant differences were observed between the RLE and Ctr groups.

Moreover, the OTA-treated group showed increased renal *i*NOS activity (*p* < 0.0001), while this activity decreased during RLE co-administration, as compared to the OTA-treated group (*p* < 0.0001). No significant differences were observed between RLE and Ctr groups (Figure 3b).

### 2.4. RLE Administration Restores Nrf2 Protein Expression

**Figure 4:**
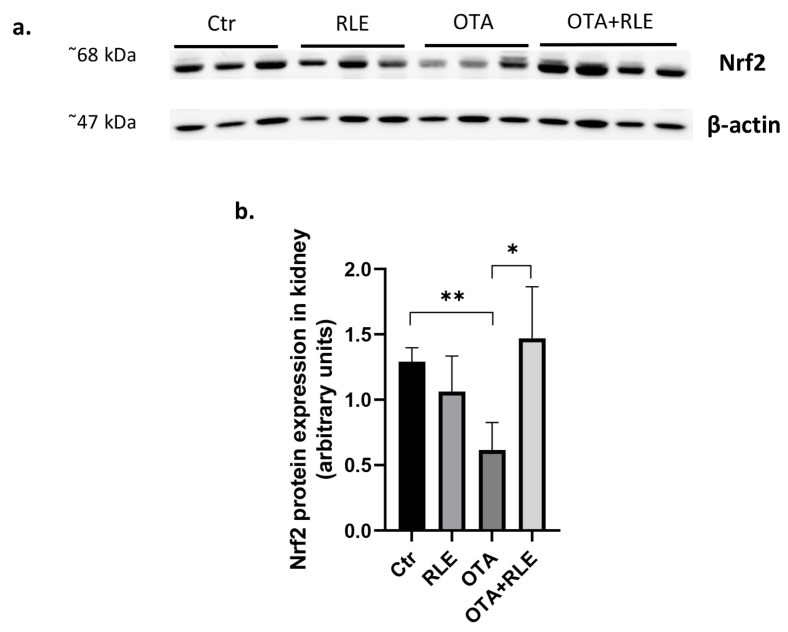
Effect of red orange and lemon extract (RLE) on Nrf2 protein expression in kidney tissues of ochratoxin A (OTA)-intoxicated rats. Control (Ctr) group; RLE group (90 mg/kg b.w.); OTA group (0.5 mg/kg), and OTA plus RLE (OTA + RLE) group. (**a**) Representative Western blot of Nrf2 in kidney tissues. (**b**) Densitometric analysis of Nrf2 in kidney tissues. The Western blots shown are the most representative of experiments conducted in triplicate. Whole blot images are in Figure S1. Data are presented as mean ± SD and were compared by an unpaired *t*-test (* *p* < 0.05, ** *p* < 0.01).

As shown in Figure 4a,b, Nrf2 expression significantly decreased in the kidney tissues of the OTA group compared to the Ctr group (*p* < 0.01). However, this value was recovered by RLE co-treatment. In fact, the latter statistically enhanced Nrf2 expression at the renal level (*p* < 0.05).

### 2.5. RLE Modulates the Inflammatory Infiltrate in the Kidney of OTA-Intoxicated Rats

**Figure 5:**
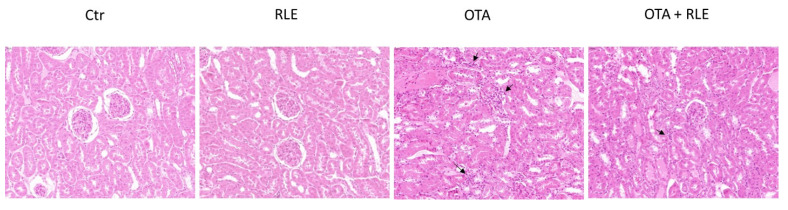
Kidney with hematoxylin and eosin (HE) staining at 40× magnification. Control (Ctr) group; red orange and lemon extract (RLE) group (90 mg/kg b.w.); ochratoxin A (OTA) group (0.5 mg/kg); and OTA plus RLE (OTA + RLE) group. OTA and OTA + RLE groups showed a moderate multifocally lymphocytic infiltrate in the interstitium (arrows).

The inflammatory alterations included a mild multifocal interstitial inflammatory infiltration that occasionally invaded Bowman’s spaces and occluded the glomeruli. Only scattered inflammatory infiltrating cells were evident in the kidneys of some in the Ctr and RLE groups (Figure 5).

Moreover, to evaluate the immunophenotype of the inflammatory cells, we assessed the immunohistochemical expression of CD3-positive T-lymphocytes, CD79-positive B cells, and Iba1-positive macrophages. The amount of CD3-positive T lymphocyte infiltration, CD79-positive B cells, and Iba1-positive macrophages were statistically increased in animals treated with OTA compared to the Ctr group (*p* < 0.01).

**Figure 6:**
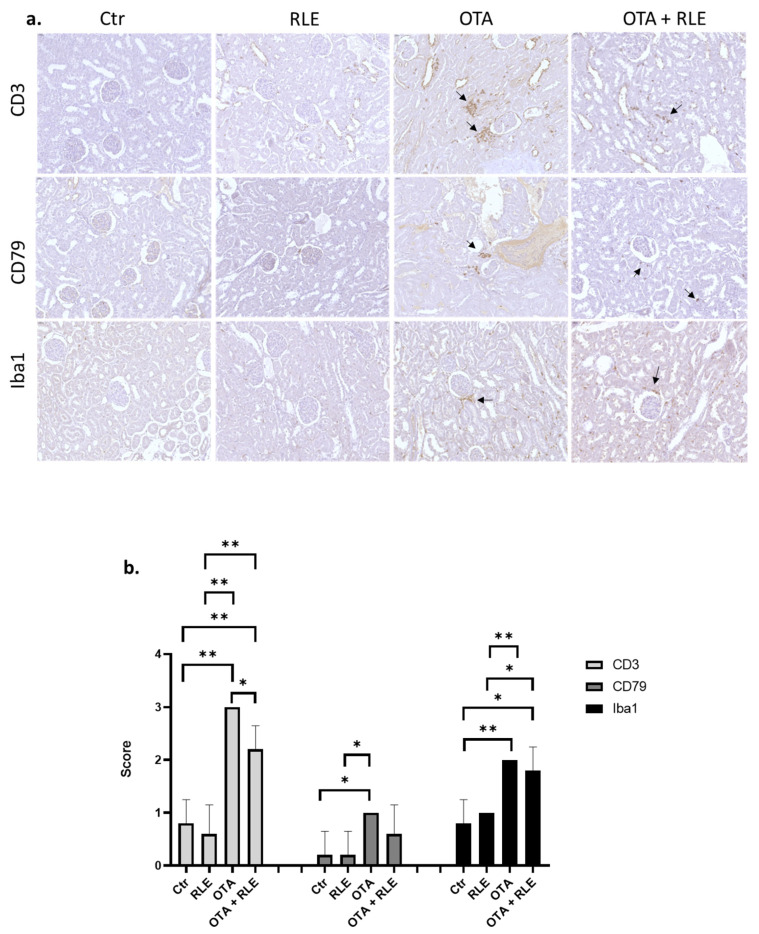
Characterization of the inflammatory infiltrates in kidneys: (**a**) Immunohistochemical characterization of inflammatory infiltrates in kidneys. Control (Ctr) group; red orange and lemon extract (RLE) group (90 mg/kg b.w.); ochratoxin A (OTA) group (0.5 mg/kg); and OTA plus RLE (OTA + RLE) group. Arrows indicate positive cells in the OTA group and OTA + RLE group. (**b**) Semi-quantitative evaluation of inflammatory infiltrates in kidneys (* *p* < 0.05, ** *p* < 0.01).

The inflammatory infiltrate was predominantly composed of CD3-positive T-lymphocytes associated with less Iba1-positive macrophages and few CD79-positive B cells in the kidneys of rats belonging to the OTA + RLE and OTA groups (Figure 6a). Although the inflammatory infiltrate score was statistically higher in the OTA and OTA + RLE groups compared to the Ctr and RLE group for each antibody (*p* < 0.05 and *p* < 0.01) (Figure 6b), we observed a significant reduction in score when comparing the OTA plus RLE group and the OTA-only group (*p* < 0.01). These data demonstrated a partial restorative effect of RLE on inflammatory damage in renal tissue.

## 3. Discussion

OTA is a natural mycotoxin normally found in feed and food that has multiple toxic effects on human and animal health. For this reason, in recent years, it has received increasing attention from various researchers intending to understand its exact mechanism of action and the most suitable methods of decontamination [27,28]. Despite extensive research carried out on the toxicity of OTA, the precise mechanism underlying its harmful effects remains uncertain. Male rats are known to be more sensitive to OTA toxicity than females, and for this reason, in our experiments, we used adult male Sprague Dawley rats treated with 0.5 mg/kg b.w. to study the common effect of OTA on OS and anti-inflammatory parameters in kidneys. Although the toxic effect of the mycotoxin has also been demonstrated in the several tissues of various animal species [29,30], the OTA target organ is the kidney [8].

The main mechanism underlying the harmful action of OTA is the involvement of OS. Exposure to OTA may be related to chronic interstitial nephropathy [31], glomerular swelling, hyperemia, and degeneration of renal tubular epithelial cells [32]. Furthermore, in our previous work, we also observed renal histopathological modifications, as well as impairment in glomerular filtration rate and serum creatinine levels in rats exposed to OTA [33]. In cell damage alteration, ROS play an important role, and antioxidant dietary supplements (such as a diet rich in fruits and vegetables with antioxidant and anti-inflammatory activities) can decrease the toxic effect of OTA and are recommended to prevent the development of chronic disease [33,34].

In this work, lipid peroxidation increased in rats treated for 14 days with oral administration of OTA, 0.5 mg/kg b.w. Previous studies have reported that the lipid peroxidation is related to the NADPH cytocrome P450 reductase [35]. OTA can induce an increase in free radical production and OS in the body. The body tries to maintain a balance of OS through antioxidant systems, e.g., SOD, CAT, and GSH enzyme activity. In this work, the oxidation parameters in the kidney tissues showed that OTA significantly decreased antioxidant activity in renal tissues compared to control group while significantly increasing the content of MDA (Figure 1). Several studies examining other animal species (such as mice and poultry) are in accordance with these results [36]. Taken together, this information shows that OTA exposure might cause free radical production and OS in the body, causing damage to lipids, proteins, and DNA. RLE treatment reduced lipid peroxidation and significantly raised antioxidant parameters, indicating that RLE extract works by restoring oxidative and antioxidant balance during OTA renal injury.

Several studies have revealed that Nrf2 protects against a wide range of disorders caused or amplified by OS, thus resulting in important protein regulation of cellular redox reactions [37]. In fact, Nrf2 controls various antioxidant enzymes’ expression and protects against OS-related illnesses [38,39,40], being downregulated during OTA contamination [36]. As ROS accumulate in the body, Nrf2 is triggered in the nucleus and binds with antioxidant reaction elements to activate the expression of antioxidant enzymes like SOD and GSH [40]. Levels of antioxidant proteins and detoxification enzymes can be modulated by Nrf2 [41]. In this work, we evidenced a significant decrease in Nrf2 protein expression in OTA-treated rats, in accordance with Loboda et al. [42]. Stachurska et al. (2013) found that OTA induced the downregulation of Nfr2 in the porcine kidney cells. Further, it was demonstrated that OTA inhibited the gene and protein expression of Nrf2 in rat kidney cells [43]. Nrf2 protein modulation has been shown to be essential for homeostasis. Thus, in fact, Nrf2-knockout animals are susceptible to various chemical insults [44]. In this work, we demonstrated that the Nrf2 pathway modulates RLE’s protective effects following OTA kidney injuries. This effect can reduce glutathione synthesis, the glutathione oxidative cycle, and oxidoreductase activity, making cells and tissues more susceptible to OS.

Data in the literature demonstrate that OTA induces inflammation [45] and that the effect of OTA on the inflammatory pathway could be a factor influencing renal disease progression and carcinogenesis [45]. Inflammatory changes in the kidneys of OTA-treated rats were confirmed in our previous article [33] mainly by the presence of mononuclear cells in the inflammatory infiltrate. In the present work, the protective effect of RLE on the inflammatory state is well demonstrated both by histopathological examination, where good restoration of inflammatory parameters was observed (Figure 5), and by immunohistochemical analysis, where we observed partial restoration of CD3+ T—lymphocytes, CD79-positive B cells, and Iba1-positive macrophages in rats treated with OTA plus RLE (Figure 6). Furthermore, a key role of RLE against the alteration of ILs and TNF-α during OTA treatment was observed (Figure 2), as well as a protective effect against macrophages (Figure 3) where, as is known, its activations promote inflammation [46]. Recent studies suggest that Nrf2 is essential for regulating the expression of proinflammatory genes and anti-inflammatory signaling [47]. Indeed, it is well demonstrated that Nrf2 activation prevents LPS-induced transcriptional upregulation of proinflammatory cytokines such as IL-6 and IL-1β in macrophages and TNF-α, IL-6, and other chemokines in neutrophils [48].

**Figure 7:**
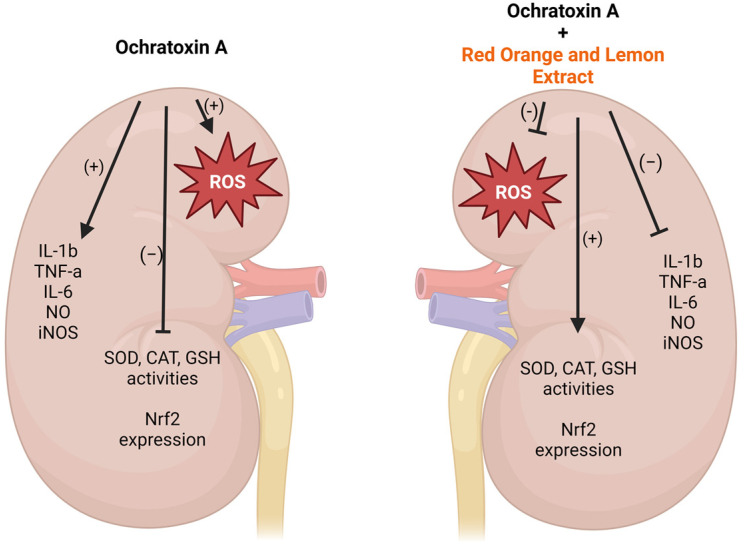
Potential mechanisms of RLE protection against OTA-induced Nrf2 inhibition. Created with Biorender.com.

Many studies have revealed Nrf2 as a transcription factor involved in OS productions [49]. It is well demonstrated that OS is characterized by an imbalance between the production of ROS and their elimination by antioxidant factors, which can lead to chronic inflammation and cause many chronic diseases [49]. Recent evidence has suggested polyphenols to be a promising natural therapy due to their potential anti-inflammatory effect linked to antioxidant activity [50]. Although future experiments are necessary to understand RLE’s exact mechanism of action on OTA-induced toxicity and it will be necessary to evaluate RLE’s suitability for secondary animals products, our findings suggest that RLE, in acting on the Nrf2 pathway, may protect against inflammation in addition to its antioxidant activity (Figure 7). Therefore, RLE could be a valid option for mitigating OTA nephrotoxicity and responding to the importance of increasing regulatory measures to mitigate nephrotoxicity induced by OTA.

## 4. Conclusions

From a One Health and public health perspective, it is very important reduce the mycotoxicosis effect of OTA using natural products with antioxidant and anti-inflammatory properties. Our results showed that RLE, with its high levels of phenols and flavonoids, can reduce OTA-induced oxidative damage and inflammation in kidneys by the activation of Nrf2 signaling pathways. The addition of RLE to feed could be a valid natural tool for reducing OTA toxicity.

## 5. Materials and Methods

### 5.1. Chemicals

OTA was purchased from Sigma-Aldrich (Milan, Italy), while the additional materials and kits used in the present work were obtained from SIAL (Rome, Italy). The Council for Agricultural Research and Economics (CREA), Research Centre for Olive, Fruit, and Citrus Crops (Acireale, Italy) produced RLE using a patented extraction technique (Italian Patent No. 102017000057761). The identification and concentrations of flavonoids and anthocyanins have been detailed in previous studies, alongside their relative compositions (%) [24].

### 5.2. Animals and Experimental Design

The guidelines of this work are in accordance with current European directives and the Italian Ministry of Health (approval number: 487/2018-PR). We studied twenty-four adult male Sprague Dawley rats, weighing 240 ± 20 g, from Charles River Laboratories (Milan, Italy). They were fed a standard diet and water ad libitum. Rats were acclimatized for 10 days prior to the experiment in constant environmental conditions at 22 ± 1 °C and normal humidity under a 12 h light/dark cycle. Animals were treated daily for 14 days by gavage as follows: (a) control (Ctr, 1 mL of sodium bicarbonate solution); (b) OTA (0.5 mg/kg b.w.) [4]; (c) RLE (90 mg/kg b.w.) [33]; (d) OTA (0.5 mg/kg b.w.) + RLE (90 mg/kg b.w.). OTA and RLE were dissolved in 1 mL solution of sodium bicarbonate. The levels of biologically active compounds of RLE were standardized and constant. The doses of OTA concentrations were chosen according to our previous experiments and the maximal residual limit imposed by European Food Safety Authority (EFSA).

### 5.3. Antioxidant Enzyme Activity and Lipid Peroxidation

At the end of experimental period, rats were sacrificed under anesthesia. Kidney tissues were promptly removed, and 0.5 g of each sample was homogenized in ice-cold phosphate buffer (pH 7.4) and then centrifuged at 3000 rpm for 20 min. The obtained supernatants were subsequently used for lipid peroxidation analysis and antioxidant enzymatic activities. In brief, we used renal tissue homogenates to assess malondialdehyde (MDA) levels via a commercially available kit from Sigma-Aldrich (Milan, Italy), and results were expressed as nmol/g of protein.

A spectrophotometer (Glomax, Promega, Milano, Italy) was used to measure SOD, CAT, and GSH activity. SOD and CAT were expressed as U/mg of protein, GSH as mg/g of protein.

### 5.4. Pro-Inflammatory Cytokines Assay

TNF-α, IL-1β, and IL-6 levels in the renal tissues were measured by commercial ELISA kits (Invitrogen, Waltham, MA, USA). The concentration of cytokines was expressed in ng/g protein.

### 5.5. Nitric Oxide (NO) Determination

Determination of NO in renal tissues was carried out with a Nitrite/Nitrate Assay Kit (Sigma-Aldrich, Milan, Italy) according to the manufacturer’s instructions. The absorbance was read at 540 nm with a spectrophotometer (Promega, Milan, Italy) and data were expressed as µmol/g tissue.

### 5.6. iNOS Activity Assay

*i*NOS activities in kidney tissues was measured by ELISA using a commercial kit according to the instructions provided by the manufacturer (Sigma-Aldrich, Milan, Italy). A polyclonal *i*NOS rat-specific antibody (Sigma-Aldrich, Milan, Italy) was used to pre-coat the microplates, and the optical densities (OD) were read using a Glomax Multi detection system. The obtained data were expressed in ng/g tissue.

### 5.7. Western Blot Assay

The Western blot assay was performed in accordance with the protocol of Damiano et al. [4]. The membrane was probed with Nfr2 (Rabbit polyclonal antibody, Antibodies.com, Stockholm, Sweden) (dilution 1:1000), and β-Actin (Mouse monoclonal antibody, Cell Signaling, Milan, Italy) (dilution 1:1000) as a housekeeping expression protein. The results were expressed as arbitrary units.

### 5.8. Histopathological Examination

Kidney samples of all groups were fixed in Bouin solution (24 h), dehydrated in ethyl alcohol, and embedded in paraffin. Sections of 4 µm were used for hematoxylin and eosin staining and immunohistochemistry procedures.

### 5.9. Immunohistochemistry

Polyclonal Rabbit Anti-CD3 (Code n. ab5690, Abcam, Cambridge, UK) at 1:200 in PBS, Monoclonal Mouse Anti-CD79 (Clone HM57, Dako, Santa Clara, CA, USA) at 1:200 in PBS, and Polyclonal Rabbit Anti-Iba1 (Code n. 019-19741, WAKO, Osaka, Japan) at 1:500 in PBS were used for the immunohistochemistry experiments. The slides were examined and acquired with Pannoramic scan II (3 Dhistech, 2023).

The degree of inflammatory infiltration for each different cell type was evaluated by counting the number of cells positive to CD3, CD79 and Iba1 antibodies using a semi-quantitative assessment scoring system [51]. The number of inflammatory cells (ICs) was scored as follows: score 0, no inflammation; mild infiltration (score 1), referring to 1 to 5 ICs per high-power field (HPF; 0.237 mm^2^; 40× objective and a 10× ocular with a field number of 22 mm); moderate infiltration (score 2), referring to 6 to 10 ICs per HPF; and severe infiltration (score 3), referring to more than 10 ICs per HPF. The average number was evaluated in at least 10 HPFs for each sample.

### 5.10. Statistical Analysis

GraphPad Prism 8 software (GraphPad Software, San Diego, CA, USA) was used for statistical analysis. Pro-inflammatory cytokines and enzymatic activities as well as lipid peroxidation were analyzed by one-way analysis of variance (ANOVA) followed by Tukey’s post-test. Western blots were analyzed using Student’s *t*-test. Regarding immunohistochemistry, semiquantitative scores were evaluated using the Mann–Whitney test. Values of *p* < 0.05 were considered statistically significant.

## Data Availability

Additional data are available from the Supplementary File.

## Supplementary Materials

The following supporting information can be downloaded at: https://www.mdpi.com/article/10.3390/toxins16030151/s1, Figure S1: (a) Whole blot of Nrf2 protein expression; (b) whole blot of β-actin protein expression.
