# Supplementary material for: Red Orange and Lemon Extract Ameliorates the Renal Oxidative Stress and Inflammation Induced by Ochratoxin A through the Modulation of Nrf2
Source: Toxins (Basel). 2024 Mar 14;16(3):151. doi: 10.3390/toxins16030151 (PMC10975592; doi:10.3390/toxins16030151)
Supplement: Supplementary file 1 [file toxins-16-00151-s001.zip › toxins-2855163-supplementary.pdf]

Red Orange and Lemon Extract Ameliorates the Renal Oxidative Stress and Inflammation Induced by Ochratoxin A through the Modulation of Nrf2

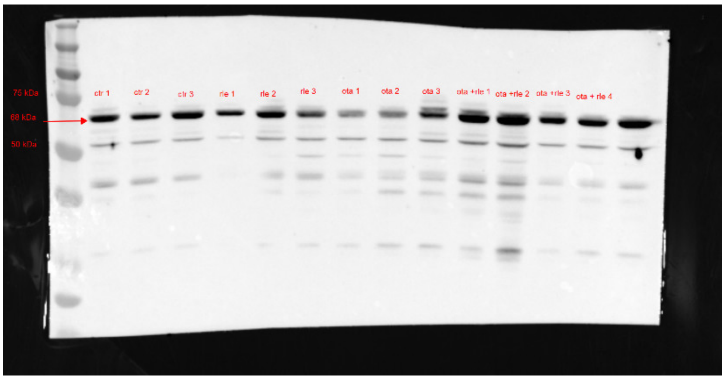

a.

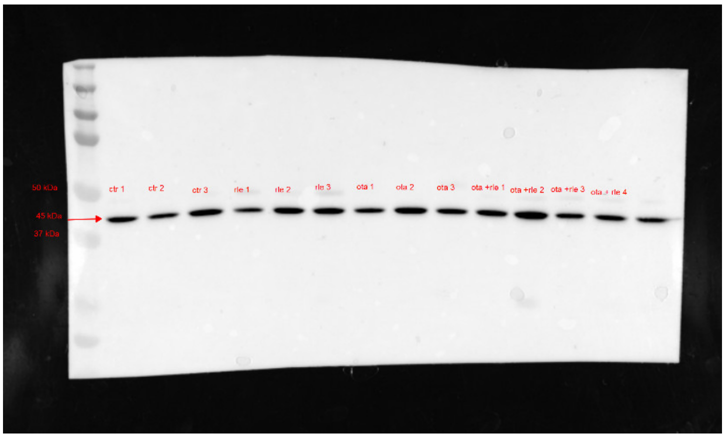

b.

Figure S1. (a) Whole blot of Nrf2 protein expression; (b) whole blot of β-actin protein expression.
